# Resilience and mental health in children and adolescents: an update of the recent literature and future directions

**DOI:** 10.1097/YCO.0000000000000741

**Published:** 2021-09-21

**Authors:** Esther Mesman, Annabel Vreeker, Manon Hillegers

**Affiliations:** Department of Child and Adolescent Psychiatry/Psychology, Erasmus MC Sophia Children's Hospital, Rotterdam, the Netherlands

**Keywords:** children and adolescents, mental health, psychopathology, resilience

## Abstract

In order to promote optimal development of children and adolescents at risk for psychiatric disorders, a better understanding of the concept resilience is crucial. Here, we provide an overview of recent work on clinical and epidemiological correlates of resilience and mental health in children and adolescents.

**Recent findings:**

Our systematic literature search revealed 25 studies that unanimously show that higher levels of resilience are related to fewer mental health problems, despite the heterogeneity of study populations and instruments. Correlates of resilience included multisystem factors, such as social, cultural, family and individual aspects, which is in line with the multisystem approach as described by recent resilience theories. Longitudinal studies are scarce but confirm the dynamical character of resilience and mental health. The application of longitudinal studies and innovative measurement techniques will improve our understanding on the cascade effects of stressors on resilience and mental health outcomes.

**Summary:**

Resilience is strongly associated with mental health in children and adolescents and deserves a more prominent role in research, prevention programs and routine clinical care. Including social, cultural and family context in the evaluation of resilience is of great value, as this can identify targets for early and preventive interventions.

## INTRODUCTION

Childhood adversity, parental psychopathology, bullying and significant threats are established risk factors for the development of psychopathology in children and adolescents [1,2^▪^,^3^–^5^]. However, the identification of risk factors does not necessarily lead to accurate prediction of psychopathology or adequate prevention. We argue that studies on emerging psychopathology should focus on factors that contribute to both risk and resilience. However, much less is known about factors that promote normative development or protect children and adolescents at risk for psychopathology. Insight into resilience and its modifiable clinical and epidemiological correlates in children and adolescents is important to inform clinicians and researchers on targets for preventive and early intervention strategies.

The purpose of this selected review is to provide an overview of recent work on resilience, its clinical and epidemiological correlates and mental health in children and adolescents. In part I we briefly describe the latest scientific insights regarding resilience and in part II we present a selected review of recent studies on resilience and mental health outcomes. We have conducted a systematic literature search (search period: January 2020–May 2021; for search strategy see Supplementary Material). Studies were included if they reported on resilience (measured with a resilience scale) [6^▪^,^7^]; mental health in children aged 0–18 years, clinical or epidemiological correlates of resilience and *N* at least 100. 

**Box 1:**
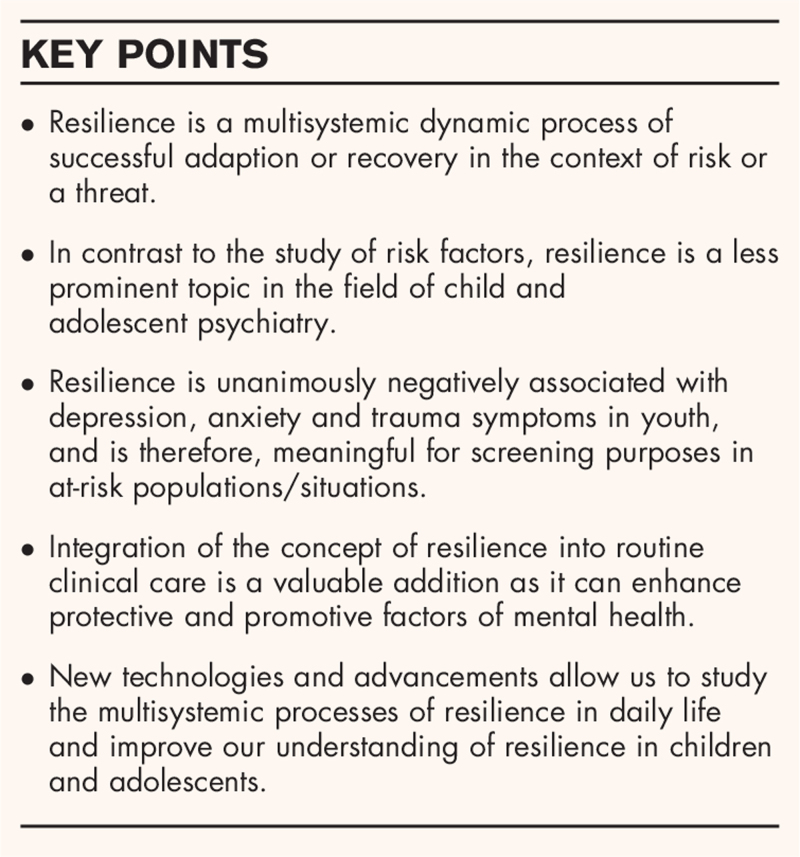
no caption available

## PART I: RESILIENCE SCIENCE

Two recent reviews describe the emergence of current theories, models, methods and challenges in the field of resilience science in relation to mental health [8^▪▪^] and from a developmental perspective [9^▪▪^]. Accordingly, resilience is best defined as a multisystemic dynamic process of successful adaption or recovery in the context of risk or a threat. In essence, there are two main components of resilience: risk or threats to the person or system (e.g. maltreatment, natural disasters, mental illness in parents) and criteria by which successful adaption or recovery is evaluated (e.g. physical health or subjective wellbeing). Both authors argue that too often mental health studies on promotive or protective factors in relation to mental health and wellbeing focus on adaptive psychological systems alone, such as coping or self-regulation, without taking into account the instability of the different physical and social ecological systems, which is referred to as the multisystem approach [8^▪▪^,^9^^▪▪^]. For an example of these multiple systems in a developmental framework, we refer to Masten's shortlist of resilience in Table 1. Additionally, apart from processes and systems of resilience at the individual, social and ecological level, also neurobiological processes, such as the hypothalamic–pituitary–adrenal axis system reflecting the biology of stress and self-regulation, are important.

**Table 1:** Masten's shortlist of multisystem resilience factors

Sensitive caregiving, close relationships, social support;
Sense of belonging, cohesion;
Self-regulation, family management, group or organization leadership;
Agency, beliefs in system efficacy, active coping;
Problem-solving and planning;
Hope, optimism, confidence in a better future;
Mastery motivation, motivation to adapt;
Purpose and a sense of meaning;
Positive views of self, family, or group;
Positive habits, routines, rituals, traditions, celebrations.

Reproduced with permission from Masten *et al.*[9^▪▪^].

Historically, assessment of resilience has been challenging. Early research focused on separate aspects of resilience, such as family support; however, an overarching concept was lacking. Current resilience measures reflect an aggregate of those aspects [9^▪▪^]; however, measures of resilience in children and adolescents are scarce [6^▪^,^7^]. Importantly, resilience is not simply the absence of psychopathology but a construct that reflects dynamic adaptation to adversity that can change over time [10]. This underscores the importance of longitudinal research. Advances in assessment methods [e.g. experience sampling methods (ESM)] and modelling (e.g. residualized approach [11], network modelling [12]) will improve our understanding of the cascade effects of a stressor in relation to resilience and mental health [9^▪▪^].

## PART II: SYSTEMATIC LITERATURE SEARCH

Our systematic search resulted in 681 studies. In total, we included 25 original studies (see Table 2). We thematically organized studies based upon population or risk. There was heterogeneity in the assessment of resilience and study populations. Four studies had a longitudinal design. Only, two studies included children with a mean age below 12 years.

**Table 2:** Overview of selected original studies

Theme	Population	Country of origin	*N*	Age range	Mean age (SD)	Study design	Resilience instrument	Outcome
General population								
Chung *et al.*[15]	Adolescents	Hong Kong	1816	11–15	NA	Cross-sectional	Resilience Scale-14	Depressive symptoms
Finch *et al.*[17]	Adolescents	Australia	456	9–14	11.54	Cross-sectional	Child and Youth Resilience	Depressive, anxiety symptoms and flourishing
Gong *et al.*[13^▪^]	Adolescents from nine schools in Wuhan	China	6019	10–17	NA	Cross-sectional	Connor-Davidson Resilience Scale	Depressive symptoms
Lee *et al.*[14^▪▪^]	Adolescents 10th–12th grade	Taiwan	450	NA	NA	Cross-sectional	Inventory of Adolescent Resilience	Depressive symptoms
Xiong *et al.*[16]	Adolescents	China	1473	12–18	15.28	Cross-sectional	Positive PsyCap Questionnaire	Anxiety/Depression
Adverse childhood experiences and negative life events								
Askeland *et al.*[26^▪^]	Population-based sample	Norway	9546	16–19	17.4	Cross-sectional	Resilience Sale for Adolescents	Depressive symptoms
Elmore *et al.*[20^▪^]	Population-based sample	USA	40 302	8–17	NA	Cross-sectional	HOPE framework, 5 independent resilient factors: child resilience, after school activities, family problem solves together, family remains hopeful, other adult factor	Depression
Hall *et al.* (2021) [19]	Youth presenting at a preventive care visit at urban academic pediatric practice	USA	450	12–18	14.9	Cross-sectional	Child Youth Resilience measure	Poor health outcome; depression, obesity, hypertension
Hamby *et al.* (2020) [23]	Community sample	USA	440	10–21	16.38	Cross-sectional	Resilience Portfolio Model	Trauma symptoms
Konaszewski *et al.*[27]	Youth sent to educational centers by a family court	Poland	253	13–18	16.3	Cross-sectional	Resilience Scale	Mental wellbeing
Kwon *et al.*[25]	Adolescent school dropouts	Korea	278	14–19	NA	Longitudinal	Ego-Resiliency scale	Depressive symptoms
Racine *et al.*[18^▪^]	Children referred to a Child Abuse Service	Canada	176	3–18	10.45	Retrospective file review	The Child and Youth Resilience Measure	Child trauma-related distress
Wei *et al.* (2021) [24]	Students from five elementary school and four middle schools	China	6510	10–17	12.6	Cross-sectional	Connor-Davidson Resilience Scale	Depressive symptoms
Zhao *et al.* (2020) [21^▪^]	Middle school students from five schools	China	742	11–19	14.32	Cross-sectional	Connor-Davidson Resilience Scale	Depression and school bullying victimization
Zhao *et al.* (2020) [22^▪^]	Left behind children recruited at two rural junior middle schools	China	345	12–17	14.31	Cross-sectional	Healthy Kids Resilience Assessment;	Depressive symptoms
Natural disasters								
An *et al.*[28^▪▪^]	Adolescents from two middle schools who were exposed to the Yancheng tornado	China	121	12–19	14.04	Longitudinal study	Connor-Davidson Resilience Scale	PTSD
Fuchs *et al.*[32]	Adolescents from Louisiana area who were exposed to both Hurricane Katrina and the Deepwater Horizon Gulf Oil Spill	USA	1268	14–18	15.6	Cross-sectional	Masten's short list for resilience	Risky substance use behavior, posttraumatic stress and depression
Liang *et al.*[31]	Students in grade 5–7 after Lushan Earth Quake	China	102	NA	12.59 ± 1.13	Longitudinal Study	Connor-Davidson Resilience Scale	Posttraumatic stress
Liu *et al.*[29^▪▪^]	Students from the most and least severe damaged school after the Wenchuan earthquake	China	1015	10–15	12.7	Longitudinal study	Block and Kremen's Ego-Resiliency Scale	Common mental health problems
Shi *et al.*[30]	Wenchuan Earthquake Adolescent Health Cohort	China	1266	NA	15.98 ± 1.28	Cross-sectional	Resilience Scale	PTSD, depression, anxiety
War								
Wilson *et al.*[33^▪^]	Palestinian refugee children living in a refugee camp	Palestina	106	11–17	NA	Cross-sectional	Child- and Youth Resilience Measure	Emotional, behavioral and psychosomatic problems
Dehnel *et al.*[34]	Syrian refugee children	Jordan	339	10–17	13.4	Cross-sectional	Child and Youth Resilience Measure	Depressive symptoms
COVID-19 pandemic								
Cusinato *et al.*[36]	Parents and children, community sample	Italy	463	5–17	NA	Cross-sectional	Child- and Youth Resilience Measure	Children's wellbeing
Yu *et al.*[37]	High school students	China	430	15–22	18.51	Cross-sectional	Brief Resilience Scale	Depression and anxiety symptoms
Wang *et al.*[38]	High school students from two schools	China	1488	12–16	13.85	Cross-sectional	Questionnaire of Adolescent Emotional Resilience	Intrusive rumination

COVID-19, coronavirus disease 2019; NA, information not available.

### General population

Five cross-sectional studies have investigated resilience in children and adolescents from the general population [13^▪^,^14^^▪▪^,^15^–^17^]. These studies showed that higher levels of resilience were related to depressive and/or anxiety symptoms. One study investigated the relationship between personality traits, resilience and depressive symptoms in 6019 high school students [13^▪^]. They found a moderating and mediating effect of resilience, and concluded that resilience may decrease the negative effect of neuroticism, and enhance the positive effect of extraversion, agreeableness and conscientiousness on depressive symptoms. Others investigated resiliency over multiple systems, such as family, school, peers [14^▪▪^,^15^,^16^]. Poorer quality in interpersonal relationships (with a parent, teacher, or peers) was related to mental health problems in children. Moreover, better interpersonal relationships were related to higher levels of resilience, which were related to fewer depressive symptoms [14^▪▪^]. Chung *et al.*[15] also found that living with a single parent was associated with lower resilience, which in turn was associated with higher levels of depression. Additionally, a study in 1473 high school students found that a construct of psychological capital (including hope, efficacy, resilience and optimism) buffered the influence of cumulative risk (an index of youth adjustment, family, school, peer and neighborhood aspects) on anxiety and depression symptoms but not life satisfaction [16]. Interestingly, a study in 456 high school students found that psychological capital was also positively related to flourishing. Furthermore, they found that the construct of psychological capital was a better predictor than the individual constructs, which shows the strength of combining different components that are related to resilience [17].

### Adverse childhood experiences and negative life events

Nine cross-sectional studies and one longitudinal study examined resilience and psychopathology in the context of adverse childhood experiences (ACE) and negative life events [18^▪^,^19^,20^▪^,21^▪^,22^▪^,^23^,^24^,^25^,26^▪^,^27^]. Overall, these studies show that higher levels of resilience are associated with mental health.

Five studies demonstrated the importance of investigating different components of resilience – for example, individual, relationship with a caregiver and educational support – in relation to ACEs and mental health in children and adolescents [18^▪^,^19^,20^▪^,^23^,^24^]. A retrospective file review in 176 children referred to a Child Abuse Service [18^▪^] found that protective factors (individual items: personal skills, peer support and social skills; caregiver: physical and psychological caregiving; and context: educational support) moderated the association between adversity and trauma-related distress. Notably, children who were exposed to higher levels of adversity had fewer protective factors. Another study in 450 youth who had experienced trauma and came to a preventive care visit, observed an inverse relationship of resilience and trauma [19] and found lower levels of resilience, in particular, relationship with caregiver, to be associated with poor health outcomes (obesity, hypertension or depression). In the National Survey of Children's Health [20^▪^] in 40 302 children, absence of child emotional resilience and lack of family problem-solving skills and hopefulness in the family, were significantly associated with depression. These effects were stronger in children experiencing more ACEs. In contrast, a study among 440 adolescents focusing on trauma symptoms, adversity and a range of psychological and social strengths, found that sense of purpose was the only unique contributor to lower trauma levels [21^▪^]. Social strengths did not add to this association.

One study reported on childhood maltreatment, resilience and gender specifically [22^▪^]. They found that the moderating effect of resilience on emotional abuse and depression was stronger in girls than boys.

Two studies investigated resilience and school-related events. Zhao *et al*[21^▪^]. studied school-bullying in 742 adolescents, and found that interpersonal relationship risks (with parents, classmates and teachers) were associated with lower individual resilience and higher risk of being bullied and depression [23]. The second study investigated 278 drop-out students over a period of 4 years [25]. They showed a dynamic course of perceived social stigma, depression and ego-resilience levels. An increase of social stigma over time was associated with higher levels of depression and lower ego-resilience. Initial and changes over time in ego-resilience mediated the relation between social stigma and depression.

One study investigated negative life events (e.g. violence from grown-ups, catastrophes, death of someone close to you), depressive symptoms and resilience in 9546 adolescents [26^▪^]. Resilient factors – that is, goal orientation, self-confidence, social competence, social support and family cohesion – were independently of life events all negatively associated with depressive symptoms, illustrating a compensatory effect. Also, a protective effect was found for goal orientation and self-confidence by showing lower depressive symptoms in adolescents who reported more negative life events.

Last, one study investigated the relationship of resilience with coping strategies in the context of negative life evens. In 253 juveniles sent to educational centers by a family court [27], resilience significantly predicted coping strategies, in particular active coping and seeking support from others. In addition, the relationship between resilience and mental wellbeing was mediated by seeking support from others and coping through discharging negative emotions.

Although these studies on resilience after ACEs and negative life events differ greatly in instruments, study design, type of adversity and outcome measures, they demonstrate that resilience is indeed a complex multisystemic dynamic process.

### Natural disasters

Five studies investigated resilience and psychopathology in children and adolescents who had experienced a natural disaster [28^▪▪^,^29^^▪▪^,^30^–^32^]. All studies showed that higher levels of resilience were related to fewer mental health problems. Two longitudinal studies will be discussed in detail [28^▪▪^,^29^^▪▪^]. In the first study, 246 adolescents were assessed 6, 9, 12 and 18 months after the Yancheng tornado [28^▪▪^]. Six to nine months after the disaster, a decline of resilience was reported, with a gradual increase after 9 months. Furthermore, individuals with lower levels of posttraumatic stress symptoms (PTSS) 6 months after the tornado reported higher levels of resilience. Individuals for whom the PTSS severity decreased more quickly in 6–12 months also reported more rapid increase in resilience in 9–18 months, which shows that resilience is dynamic and takes time. Recovery of PTSS was associated with a steeper increase of resilience. Looking at timing of recovery, changes in PTSS and resilience occurred just after restoration of schools and homes, underscoring the importance of the multisystemic approach. The second study investigated the relationship between self-esteem, resilience, social support and mental health in 1015 adolescents 2 years after the Wenchuang earthquake [29^▪▪^]. Self-esteem and common mental health problems had a mutual negative effect on each other, whilst self-esteem and resilience positively affected each other. Social support had a promoting effect on self-esteem and resilience and a buffering effect against mental health problems. Moreover the study illustrated that all these aspects fluctuated over time. Other cross-sectional studies found that maternal parenting styles were significant predictors of resilience after earthquake experiences [30]; a positive association between PTSS, creative thinking and resilience were possible manifestations of posttraumatic growth [31]. Moreover, one study found that resilience mediated the association between depression and risky substance use behavior after natural disasters [32].

### War

Two cross-sectional studies reported on resilience in children and adolescents living in refugee camps [33^▪^,^34^]. One study illustrated that resilience among 106 children and adolescents in refugee camps was positively associated with perceived level of community support, spiritual, cultural and educational resources [33^▪^]. The authors underscore that in addition to a universal approach in resiliency programs, the context must be prioritized as well. Another study in 339 children and adolescents reported that higher levels of resilience were related to fewer symptoms of depression but that prior trauma was not associated with resilience [34], which may reflect the dynamic process of resilience.

### Coronavirus disease 2019 pandemic

The coronavirus disease 2019 (COVID-19) pandemic is a global threat for public mental health. The pandemic exposes the underlying causes of insecurity, social and economic consequences, and thus challenges the multiple systems contributing to resilience [35]. Indeed, there appears a steep rise in mental health problems in youth [2^▪^]. Thus far, three cross-sectional studies have investigated resilience and mental wellbeing among children and adolescents during the pandemic [36–38]. An Italian study in 463 families during the first COVID-19 wave found a positive association between the child's resilience and wellbeing. Also more parental stress was related to lower levels of resilience in offspring [36]. Another study among 430 Chinese adolescents and the perceived impact of the pandemic showed both psychological distress and posttraumatic growth. Resilience and meaning in life served as protective factors for mental health [37]. Furthermore, in a study in 1488 Chinese teenagers, creative ideational behavior was positively related to intrusive rumination during the pandemic [38]. Interestingly, like Liang *et al.*[31], resilience and creativity were associated. In this study, the relationship between creativity and intrusive rumination was stronger in students with low levels of emotional resilience as compared with students with high levels of emotional resilience.

## FUTURE DIRECTIONS

In this selected review, we discussed the recent literature on resilience, mental health and its correlates in children and adolescents up to 18 years old. Despite the heterogeneity between studies because of differences in instruments and study populations, all studies consistently show that higher levels of resilience are related to fewer mental health problems. Moreover, multiple factors, such as personal skills, social skills, peer support, school environment, contact with peers, parent–child relationship, family problem-solving, parental resilience, parental stress and goal orientation were related to resilience and demonstrate the importance of a multisystemic approach including social, cultural and family context in the study of resilience.

This review underscores that the construct of resilience is essential in the study of risk for psychopathology. However, the majority of the studies in this review were cross-sectional and did not measure resilience or psychopathology over time. As resilience is a dynamic process, longitudinal studies are essential [10]. Therefore, we argue that prospective studies on the influence of both risk and resilience in children and adolescents are needed.

The study of resilience can inform researchers and clinicians on targets for screening, intervention and preventive strategies. As higher levels of resilience are clearly associated with wellbeing in the child, and the context plays a significant role, we strongly encourage to incorporate the assessment of resilience in standard clinical care. Children and families with lower levels of resilience may be in need of other therapeutic support and more close monitoring compared with those with higher levels of resilience and a stable and supporting environment.

Over the past two decades, our research group has extensively studied intergenerational transmission of psychopathology in offspring of patients with severe mood disorders and psychotic disorders [1,3,39]. To advance our understanding of the development of psychopathology and to accommodate the growing need from clinicians and patients’ organizations for tools to promote resilience and increase wellbeing in children and adolescents, our mission has expanded from identification of risk factors of psychopathology to understanding how protective factors are related to wellbeing and daily life functioning. This has resulted in the development of new, online preventive intervention tools for children and adolescents at increased risk of psychopathology. An example of this is the Grow it! App, a gamified smartphone application, which monitors emotions, thoughts and behaviors in daily life (using ESM), and offers daily challenges using cognitive behavior therapy-based elements to promote adaptive coping. During the COVID-19 pandemic, Grow it! has been offered to individuals from the general population (aged 12–25 years) [40]. Currently, we are also studying the efficacy of this application in offspring of parents with mood disorders, and children with a chronic illness.

## CONCLUSION

Resilience is a crucial aspect in the study of psychopathology in children and adolescents and should be studied in a multisystem approach, including individual, social, familial and cultural context. As resilience is a dynamic process that can change over time, there is a need for longitudinal studies that assess resilience and psychopathology in children and adolescents prospectively. An improved understanding of resilience factors might offer new targets both for clinical settings and for preventive and early intervention programs.

## Acknowledgements

*The authors would like to express their gratitude towards Sabrina Meertens-Gunput from the Erasmus MC Medical Library for developing the search strategy. We thank Fleur Helmink, MSc for her contributions to the manuscript.*

### Financial support and sponsorship

*The current work was supported by the Netherlands Organization for Health Research and Development (ZonMw), project number: 606360098021 and 636320009.*

### Conflicts of interest

*There are no conflicts of interest.*

## Supplementary Material

Supplemental Digital Content

## REFERENCES AND RECOMMENDED READING

Papers of particular interest, published within the annual period of review, have been highlighted as:

▪ of special interest
▪▪ of outstanding interest
