# Supplementary material for: Resilience and mental health in children and adolescents: an update of the recent literature and future directions
Source: Curr Opin Psychiatry. 2021 Sep 21;34(6):586–92. doi: 10.1097/YCO.0000000000000741 (PMC8500371; doi:10.1097/YCO.0000000000000741)
Supplement: Supplemental Digital Content [file coip-34-586-s001.docx]

**Supplementary material - Search Strategy and results per database**

**Date of search: 5 may 2021**

| *Database* | *Records* | *Records after duplicates removed* |
| --- | --- | --- |
| Embase.com | 455 | 454 |
| Medline (Ovid) | 236 | 65 |
| PsycINFO (Ovid) | 170 | 49 |
| Web of Science | 256 | 113 |
| Cochrane Central | 6 | 0 |
| **Total** | **1123** | **681** |

**Embase.com 455**

('psychological resilience'/de OR (resilienc*):ab,ti,kw) **AND** ('mental disease'/exp/mj OR (((mental*) NEAR/3 (disease* OR illness* OR disorder*)) OR psychopatholog*):ab,ti,kw) **AND** (child/mj/exp OR adolescent/mj/exp OR adolescence/mj/exp OR 'child behavior'/mj/de OR 'child parent relation'/mj/de OR pediatrics/mj/exp OR childhood/mj/exp OR 'child nutrition'/mj/de OR 'infant nutrition'/mj/exp OR 'child welfare'/mj/de OR 'child abuse'/mj/de OR 'child advocacy'/mj/de OR 'child development'/mj/de OR 'child growth'/mj/de OR 'child health'/mj/de OR 'child health care'/mj/exp OR 'child care'/mj/exp OR 'childhood disease'/mj/exp OR 'child death'/mj/de OR 'child psychiatry'/mj/de OR 'child psychology'/mj/de OR 'pediatric ward'/mj/de OR 'pediatric hospital'/mj/de OR 'pediatric anesthesia'/mj/de OR 'pediatric intensive care unit'/mj/de OR 'neonatal intensive care unit'/mj/de OR 'prematurity'/mj/de OR (adolescen* OR preadolescen* OR infan* OR newborn* OR (new NEXT/1 born*) OR baby OR babies OR neonat* OR prematur* OR pre-matur* OR child* OR kid OR kids OR toddler* OR teen* OR boy* OR girl* OR minors OR underag* OR (under NEXT/1 (age* OR aging OR ageing)) OR juvenil* OR youth* OR kindergar* OR puber* OR pubescen* OR prepubescen* OR prepubert* OR pediatric* OR paediatric* OR school* OR preschool* OR highschool* OR suckling* OR PICU OR NICU OR PICUs OR NICUs):ab,ti,kw) AND [2020-2021]/py NOT [conference abstract]/lim

**Medline (Ovid) 236**

(Resilience, Psychological/ OR (resilienc*).ab,ti,kf.) **AND** (exp * Mental Disorders/ OR (((mental*) ADJ3 (disease* OR illness* OR disorder*)) OR psychopatholog*).ab,ti,kf.) **AND** (exp * Child/ OR exp * Infant/ OR exp *Adolescent/ OR exp *"Child Behavior"/ OR exp *"Parent Child Relations"/ OR exp * "Pediatrics"/ OR * "Child Nutrition Sciences"/ OR * "Infant nutritional physiological phenomena"/ OR exp * "Child Welfare"/ OR * "Child Development"/ OR exp * "Child Health Services"/ OR exp * "Child Care"/ OR * "Child Rearing"/ OR exp * "Child development Disorders, Pervasive"/ OR * "Child Psychiatry"/ OR * "Child Psychology"/ OR * "Hospitals, Pediatric"/ OR exp * "Intensive Care Units, Pediatric"/ OR (adolescen* OR infan* OR newborn* OR (new ADJ born*) OR baby OR babies OR neonat* OR prematur* OR pre-matur* OR child* OR kid OR kids OR toddler* OR teen* OR boy* OR girl* OR minors OR underag* OR (under ADJ1 (age* OR aging OR ageing)) OR juvenil* OR youth* OR kindergar* OR puber* OR pubescen* OR prepubescen* OR prepubert* OR pediatric* OR paediatric* OR school* OR preschool* OR highschool* OR suckling* OR PICU OR NICU OR PICUs OR NICUs).ab,ti,kf.) NOT (letter* OR news OR comment* OR editorial* OR congres* OR abstract* OR book* OR chapter* OR dissertation abstract*).pt.

2.

Limit 1 to yr=2020-2021

**PsycINFO (Ovid) 170**

("Resilience (Psychological)"/ OR (resilienc*).ab,ti.) **AND** (exp * Mental Disorders/ OR (((mental*) ADJ3 (disease* OR illness* OR disorder*)) OR psychopatholog*).ab,ti.) **AND** ((100 OR 200).ag. OR (adolescen* OR infan* OR newborn* OR (new ADJ born*) OR baby OR babies OR neonat* OR prematur* OR pre-matur* OR child* OR kid OR kids OR toddler* OR teen* OR boy* OR girl* OR minors OR underag* OR (under ADJ1 (age* OR aging OR ageing)) OR juvenil* OR youth* OR kindergar* OR puber* OR pubescen* OR prepubescen* OR prepubert* OR pediatric* OR paediatric* OR school* OR preschool* OR highschool* OR suckling* OR PICU OR NICU OR PICUs OR NICUs).ab,ti.) NOT (letter* OR news OR comment* OR editorial* OR congres* OR abstract* OR book* OR chapter* OR dissertation abstract*).pt.

2.

Limit 1 to yr=2020-2021

**Web of Science 256**

TS=(((resilienc*)) **AND** ((((mental*) NEAR/2 (disease* OR illness* OR disorder*)) OR psychopatholog*)) **AND** ((adolescen* OR preadolescen* OR infan* OR newborn* OR (new NEAR/1 born*) OR baby OR babies OR neonat* OR prematur* OR pre-matur* OR child* OR kid OR kids OR toddler* OR teen* OR boy* OR girl* OR minors OR underag* OR (under NEAR/1 (age* OR aging OR ageing)) OR juvenil* OR youth* OR kindergar* OR puber* OR pubescen* OR prepubescen* OR prepubert* OR pediatric* OR paediatric* OR school* OR preschool* OR highschool* OR suckling* OR PICU OR NICU OR PICUs OR NICUs))) AND DT=(Article OR Review OR Early Access) AND PY=(2020-2021)

**Cochrane Central 6**

((resilienc*):ab,ti,kw) **AND** ((((mental*) NEAR/3 (disease* OR illness* OR disorder*)) OR psychopatholog*):ab,ti,kw) **AND** ((adolescen* OR preadolescen* OR infan* OR newborn* OR (new NEXT/1 born*) OR baby OR babies OR neonat* OR prematur* OR pre NEXT matur* OR child* OR kid OR kids OR toddler* OR teen* OR boy* OR girl* OR minors OR underag* OR (under NEXT/1 (age* OR aging OR ageing)) OR juvenil* OR youth* OR kindergar* OR puber* OR pubescen* OR prepubescen* OR prepubert* OR pediatric* OR paediatric* OR school* OR preschool* OR highschool* OR suckling* OR PICU OR NICU OR PICUs OR NICUs):ab,ti,kw)
